# Impact of viral telomeric repeat sequences on herpesvirus vector vaccine integration and persistence

**DOI:** 10.1371/journal.ppat.1012261

**Published:** 2024-05-28

**Authors:** Caroline Denesvre, Yu You, Sylvie Rémy, Tereza Vychodil, Katia Courvoisier, Zoltán Penzes, Luca D. Bertzbach, Ahmed Kheimar, Benedikt B. Kaufer

**Affiliations:** 1 INRAE, UMR1282 ISP, Equipe Biologie des Virus Aviaires, Nouzilly, France; 2 Institute of Virology, Freie Universität Berlin, Berlin, Germany; 3 Ceva Santé Animale, Ceva-Phylaxia, Budapest, Hungary; 4 Leibniz Institute of Virology (LIV), Department of Viral Transformation, Hamburg, Germany; 5 Department of Poultry Diseases, Faculty of Veterinary Medicine, Sohag University, Sohag, Egypt; 6 Veterinary Centre for Resistance Research (TZR), Freie Universität Berlin, Berlin, Germany; University of Wisconsin-Madison, UNITED STATES

## Abstract

Marek’s disease virus (MDV) vaccines were the first vaccines that protected against cancer. The avirulent turkey herpesvirus (HVT) was widely employed and protected billions of chickens from a deadly MDV infection. It is also among the most common vaccine vectors providing protection against a plethora of pathogens. HVT establishes latency in T-cells, allowing the vaccine virus to persist in the host for life. Intriguingly, the HVT genome contains telomeric repeat arrays (TMRs) at both ends; however, their role in the HVT life cycle remains elusive. We have previously shown that similar TMRs in the MDV genome facilitate its integration into host telomeres, which ensures efficient maintenance of the virus genome during latency and tumorigenesis. In this study, we investigated the role of the TMRs in HVT genome integration, latency, and reactivation *in vitro* and *in vivo*. Additionally, we examined HVT infection of feather follicles. We generated an HVT mutant lacking both TMRs (vΔTMR) that efficiently replicated in cell culture. We could demonstrate that wild type HVT integrates at the ends of chromosomes containing the telomeres in T-cells, while integration was severely impaired in the absence of the TMRs. To assess the role of TMRs *in vivo*, we infected one-day-old chickens with HVT or vΔTMR. vΔTMR loads were significantly reduced in the blood and hardly any virus was transported to the feather follicle epithelium where the virus is commonly shed. Strikingly, latency in the spleen and reactivation of the virus were severely impaired in the absence of the TMRs, indicating that the TMRs are crucial for the establishment of latency and reactivation of HVT. Our findings revealed that the TMRs facilitate integration of the HVT genome into host chromosomes, which ensures efficient persistence in the host, reactivation, and transport of the virus to the skin.

## Author summary

Several herpesviruses harbor telomeric repeats (TMRs) at the ends of their genomes. These TMRs are identical to the host telomeres, which protect the ends of host chromosomes from deterioration. One of these viruses is the herpesvirus of turkey (HVT), which is commonly used as a live-attenuated vaccine against MDV but also as a vaccine vector. Our study revealed that HVT efficiently integrates into the ends of host chromosomes containing the telomeres. Removal of the TMRs from the virus genome did not affect virus replication but severely impaired integration. In addition, persistence of the vaccine virus was drastically reduced in the host. Our data also revealed that integration and/or persistence of the virus is crucial for the transport of the virus to the skin of infected animals, where HVT replicates and is shed into the environment. Our study provides important insights into HVT biology and sheds light on the role of the TMRs in virus integration, persistence and shedding.

## Introduction

Over the past decades, the global poultry industry has experienced considerable growth to meet the growing demand for animal protein. However, this expansion with the adoption of intensive farming practices resulted in a strong selective pressure on a number of pathogens. Protection against the major poultry pathogens is achieved by increased biosecurity, hygienic measures, and vaccination. Historically, the turkey herpesvirus (HVT, Mardivirus meleagridalpha 1, MeAHV1) was the first widely used vaccine to protect against the highly oncogenic Marek’s disease virus (MDV, Mardivirus gallidalpha 2, GaAHV2) [1]. HVT belongs to the genus *Mardivirus* in the *Alphaherpesvirus* subfamily and shares genetic, serological, and biological properties with MDV. Aside from the protection against MDV, HVT has been extensively used as a vector vaccine encoding antigens for other pathogens including infectious bursal disease, Newcastle disease (ND), avian influenza, and infectious laryngotracheitis [2–10]. Using HVT as a vaccine vector for the generation of these recombinant vaccines offers several advantages: i) HVT has a large coding capacity and can harbor multiple foreign antigens [11,12]; ii) administration of a single dose provides long-term protection [13] and iii) HVT recombinant vaccines are often safer than conventional vaccines (*e*.*g*. for infectious laryngotracheitis) [9,14]. Over the last three decades, the use of HVT recombinant vaccines in poultry industry has immensely increased across the globe.

Like all herpesviruses, HVT establishes latency allowing the vaccine virus to persist in the host [15–18]. Most herpesviruses maintain their genome in the nucleus of latently infected cells in the form of a circular extrachromosomal DNA, termed episome (e.g. herpes simplex virus 1, varicella-zoster virus, Epstein-Barr virus, etc.) [17,19]. Interestingly, several herpesviruses including MDV and human herpesvirus 6A (HHV-6A) and 6B (HHV-6B), were previously shown to integrate their genome into the telomeres of latently infected cells [20–24]. However, hardly anything is known about how HVT persists in vaccinated chickens.

HVT has a double-stranded DNA class E genome of approximately 160 kbp [25]. The genome harbors viral telomeric repeat arrays (TMRs) at both ends of the linear genome as well as in the internal repeat long and short (IR_L_-IR_S_) junction [23]. Each HVT a-like sequence possesses a single TMR array, while the MDV counterpart contains two, the multiple telomeric repeats (mTMRs) and short telomeric repeats (sTMRs). Previous research has demonstrated that the TMRs are dispensable for MDV lytic replication, but play a crucial role in MDV integration, tumorigenesis, and reactivation [21,26]. Until now, the role of the TMRs in the HVT genome remains elusive.

To elucidate the role of the TMRs in the HVT genome, we generated a mutant lacking its TMRs (vΔTMR). We established an *in vitro* integration assay and assessed the integration frequency of both wild type (WT) and the mutant virus. Our data revealed that WT HVT efficiently integrates into the ends of host chromosomes, while integration was severely impaired in the absence of the TMRs. In addition, we investigated the role of the TMRs in HVT replication and latency in vaccinated chickens. Deletion of the TMRs significantly reduced vΔTMR genome persistence in the blood and spleens, but also transport of the virus to the feather follicle epithelium (FFE). Taken together, our data revealed that the TMRs are crucial for HVT genome integration, persistence, and thus, reactivation in the vaccinated host.

## Results

### The TMRs of HVT are dispensable for vaccine virus replication *in vitro*

**Fig 1:**
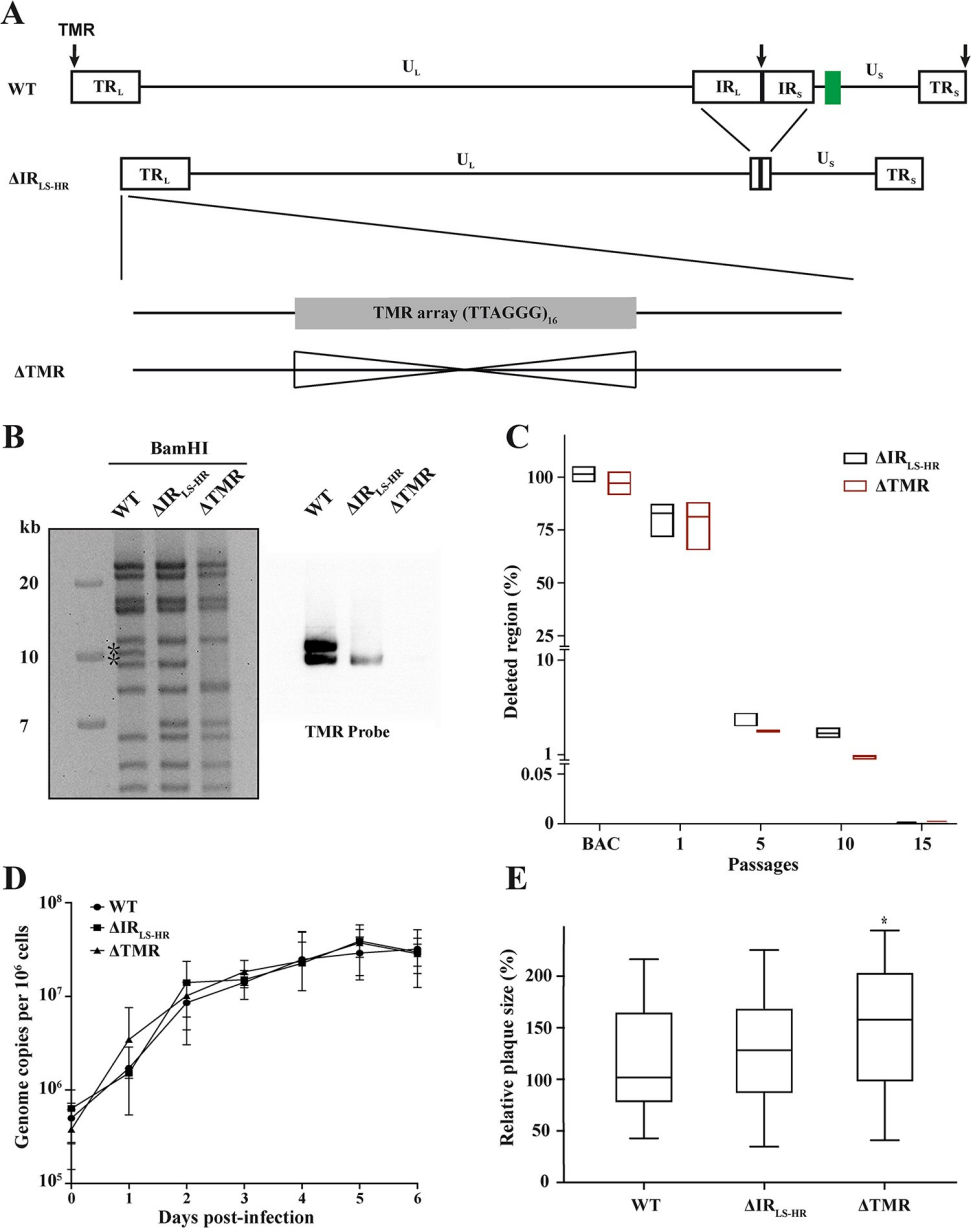
Generation and characterization of the HVT TMR deletion virus *in vitro*. (A) Overview of the HVT genome with a focus on the a-like regions containing TMRs (black arrows). The mini-F cassette with its pTK-eGFP reporter cassette is shown as a green square. Unique long and short regions (U_L_ and U_S_) are flanked by terminal and internal repeat long and short regions (TR_L_, TR_S_, IR_L_, and IR_S_). First, most of the IR_L_ and IR_S_ were deleted (ΔIR_LS-HR_) followed by the deletion of TMR arrays in the TR region (ΔTMR). (B) RFLP analysis with BamHI (TMR-containing fragments are indicated with asterisks) and Southern blotting of WT and indicated mutant BACs. A TMR-specific probe was used to detect the presence of the TMRs. (C) qPCR analysis of the IR_LS_ deletion site upon reconstitution of the indicated viruses and passages. BAC DNA of the respective viruses was used as a control. The ratio of the deleted region relative to the viral genome is shown in percentages and as boxes indicating both the means and the range between minimum and maximum values (*n* = 3). (D) Multi-step growth kinetics of the indicated viruses. Mean viral genome copies per one million cells are shown for the indicated time points (p>0.05, Kruskal-Wallis test, *n* = 3, error bars indicate standard deviations (SD)). (E) Plaque size assays of the indicated viruses. The plaque diameter relative to the WT is shown as box plots with median, minimum, and maximum. *, p<0.05, Kruskal-Wallis test (n = 50).

To assess the role of the TMRs in HVT integration and persistence, we deleted the entire TMR arrays (96 bp) within the a-like sequences from the infectious BAC clone of the HVT vaccine strain FC126, encoding an eGFP in the mini-F sequences (Fig 1A). This was achieved by first deleting most of the IR_LS_ region containing one of the TMR (vΔIR_LS-HR_; ‘LS’ stands for the long and short repeats and ‘HR’ for homologous recombination) to establish a platform virus allowing the easy manipulation of the repeat regions, and subsequently deleting the remaining TMR copy [27]. We previously demonstrated that such a deletion of the IR_LS_ is rapidly restored by copying the remaining repeat upon virus reconstitution in the MDV genome [27]. To confirm the deletion of the TMRs, the resulting BAC clones were screened by PCR, Sanger sequencing, restriction fragment length polymorphism (RFLP), Southern blotting (Fig 1B), and next generation sequencing (NGS). Southern blotting revealed that the TMRs were deleted in the consecutive rounds of mutagenesis and that the final clone lacks all TMRs. To address whether the deleted IR_LS_ region is efficiently restored upon reconstitution, we propagated the viruses for 15 passages and assessed the deletion site by qPCR. Over 95% of the viral genomes restored the deleted region already at passage 5, indicating that the IR_LS_ deletion in vΔIR_LS-HR_ and vΔTMR is rapidly restored for HVT (Fig 1C) as previously observed for MDV [27].

To determine if the deletion of the TMRs affects virus replication, we assessed the replication properties of the recombinant virus. Multi-step growth kinetics revealed that vΔIR_LS-HR_ and vΔTMR replicate comparable to WT (Fig 1D). Efficient replication and cell-to-cell spread were also observed in plaque size assays, where vΔTMR even replicated significantly better than the WT (Fig 1E). Taken together, our data revealed that the IR_LS_ are rapidly restored and that the TMRs do not play a role in HVT replication.

### HVT efficiently integrates into the ends of host chromosomes, which is facilitated by the viral TMRs

**Fig 2:**
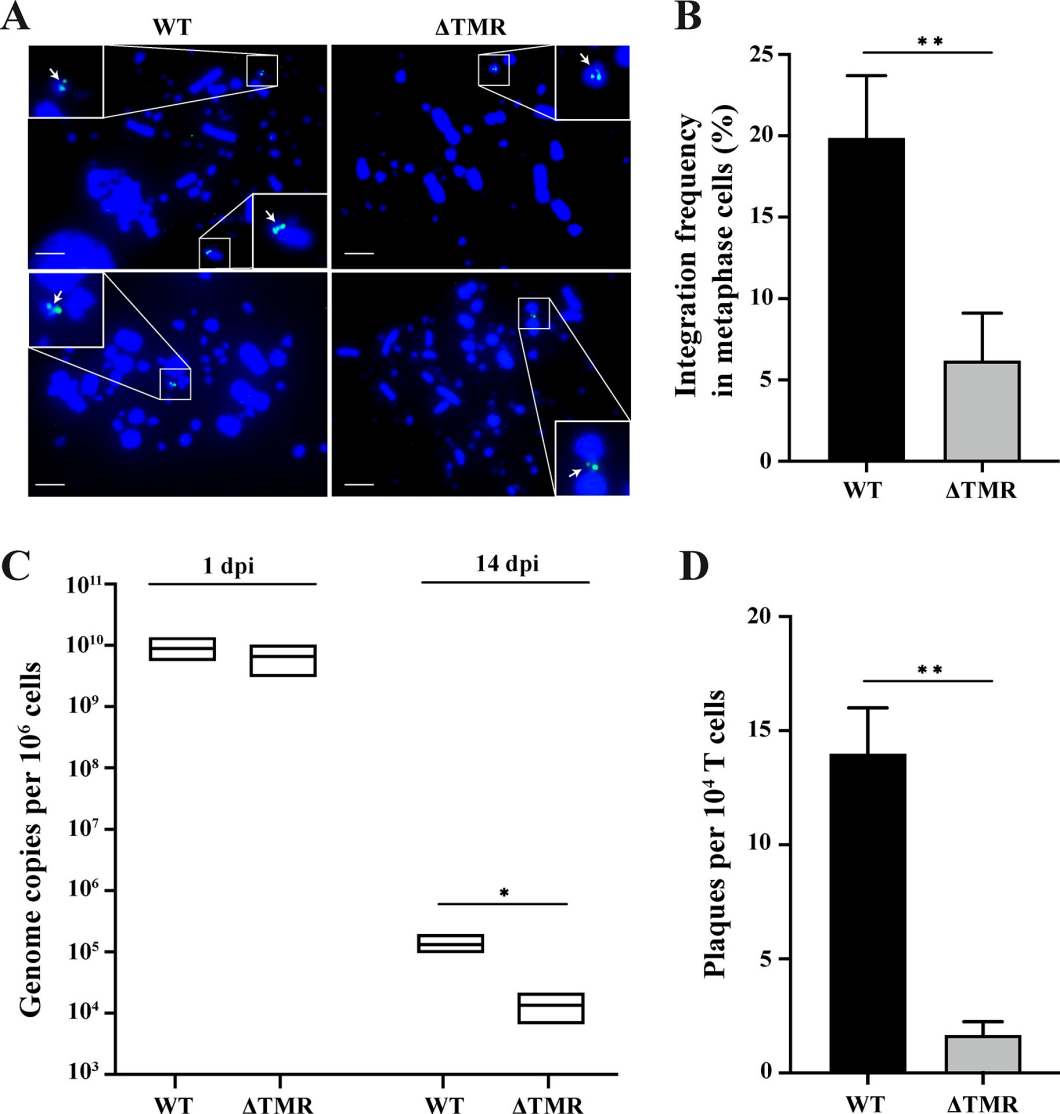
The absence of the TMRs impairs HVT integration and genome maintenance *in vitro*. (A) FISH analysis detecting the HVT genome in chromosomes of latently T-cells infected cells. Representative images of metaphase chromosomes (DAPI, blue) and the integrated HVT genome (FITC, green) are shown for the indicated viruses. Small white arrows indicate the location of HVT genomes at the chromosome ends (WT; left images) or elsewhere in the chromosome (vΔTMR; right images). The scale bars correspond to 10 μm. (B) Integration frequency of the indicated viruses was quantified in at least 100 random metaphases per experiment. Results are shown as the mean of three independent experiments with SDs (**, p<0.01, t-test). (C) HVT genome maintenance was quantified by assessing virus genome copies at 1 dpi and 14 dpi. Significant differences between WT and vΔTMR are indicated with an asterisk (*, p<0.05, Mann-Whitney U-test). Results are shown as the mean of three independent experiments, as boxes indicating both the means and the range between minimum and maximum values. (D) Reactivation efficiency in latently infected T-cells. The data are shown as the mean number of plaques per 10^4^ T-cells upon co-cultivation with CECs (**, p<0.01, t-test, *n* = 3). The error bars indicate the SDs.

To investigate the integration of HVT and the role of the TMRs in this process, we established an *in vitro* integration assay using 855–19 T-cells as previously described for MDV [26]. Fluorescence *in situ* hybridization (FISH) analyses revealed that HVT can efficiently integrate at the end of one or multiple chromosomes in about 20% of the infected 855–19 T-cells [24]. In contrast, integration efficiency was significantly reduced in the absence of the TMRs (Fig 2A and 2B). Intriguingly, in the absence of the TMRs, integration did not appear to be at the ends of the chromosomes as previously observed for MDV lacking TMRs (Fig 2A) [21,22,26]. Consistently, genome maintenance of the HVT vΔTMR was significantly impaired (9.8-fold) compared to the WT at 14 days post-infection (dpi), indicating that the TMRs play an important role in the maintenance of the latent HVT genome (Fig 2C).

As HVT frequently reactivates and is shed from the skin, we examined the reactivation properties of the integrated WT and mutant HVT in latently infected T-cells. Strikingly, reactivation of the vΔTMR was significantly impaired (8.4-fold) compared to the WT (Fig 2D). Taken together, our data revealed that HVT integrates efficiently in latently infected T-cells and that the TMRs play an important role in the integration process and genome maintenance, resulting in a lower reactivation frequency.

### The absence of the TMRs reduces viral load in the PBMCs of infected animals

**Fig 3:**
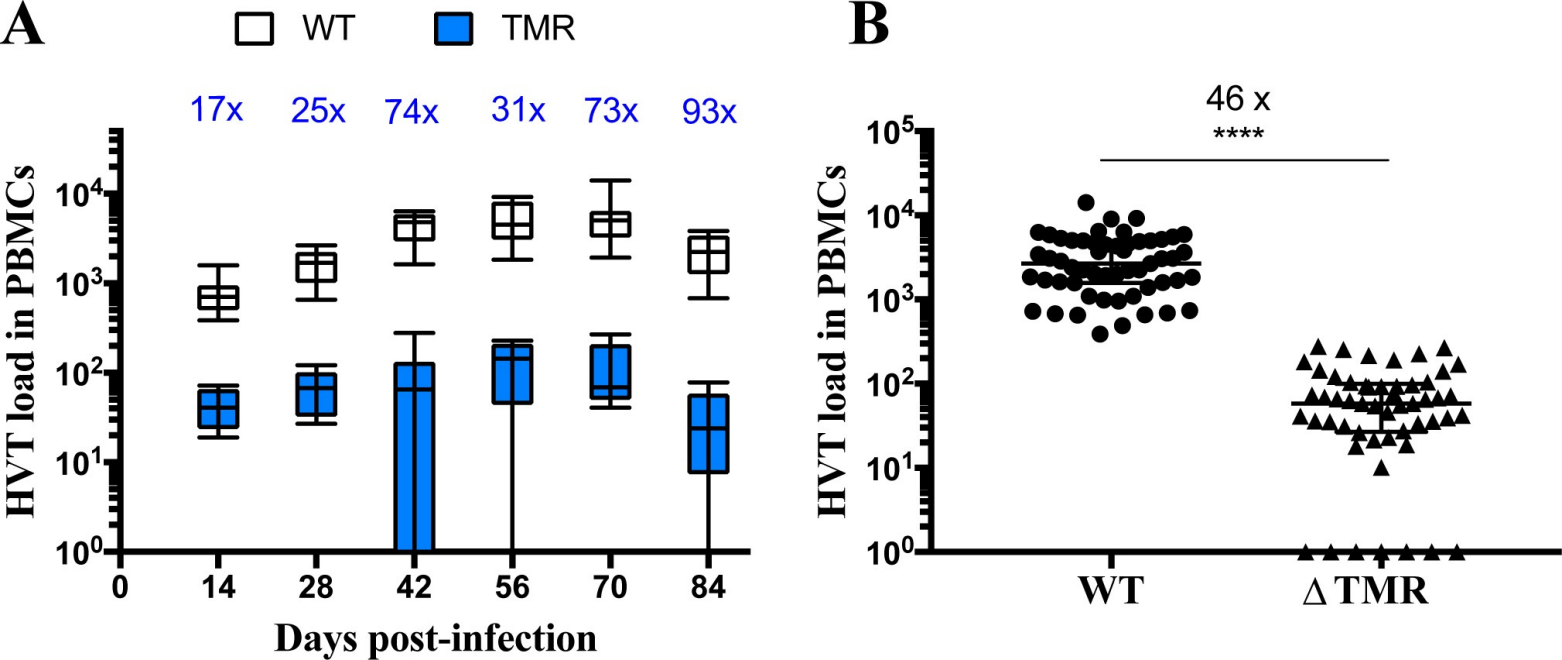
The absence of the TMRs severely reduces the HVT loads in PBMCs of infected chickens. HVT genomes were quantified in PBMCs of chickens infected with indicated viruses by qPCR. (A) Dynamic of HVT load over time. Data are shown in Tukey boxes (WT in black; vΔTMR in blue). The median loads were reduced at all time points (vΔTMR vs. WT; p<0.0001, Wilcoxon test with a Holm correction for multiple comparisons). The fold-changes are indicated in blue above each box. (B) HVT load across time for both viruses. Data are shown with each HVT load visible as a dot. The load medians are visible as long horizontal bars (2662 genome copies/one million cells for WT vs 58 for vΔTMR) (****, p<0.0001, Mann-Whitney test, two-tailed).

To assess the role of the TMRs *in vivo*, we subcutaneously inoculated one-day-old chickens with 5000 pfu of either vΔTMR or WT HVT (the parental virus). First, we quantified viral genome copies in peripheral mononuclear cells (PBMCs) of the infected animals by qPCR over time, as the virus likely integrates into the chromosomes of these cells. The vΔTMR genome copies were significantly reduced in PBMCs between 17- to 93-fold from14 to 84 dpi compared to the WT (Fig 3A). For both viruses, virus loads peaked around 56 dpi (Fig 3A). Moreover, 13.2% of the PBMC samples of the vΔTMR group had no detectable viral genome copies, while 100% of the WT samples were positive for the virus (Fig 3B). Regardless of the time, viral loads of the vΔTMR virus in PBMCs were significantly reduced (46-fold) compared to the WT (Fig 3B). Taken together, our data revealed that the HVT genome copies were significantly reduced in PBMCs in the absence of TMRs.

### The absence of TMRs severely impairs viral latency

**Fig 4:**
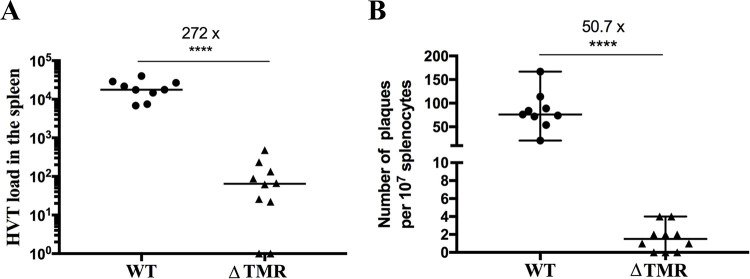
HVT maintenance and reactivation are severely impaired in the spleen in the absence of the TMRs. (A) HVT genomes were quantified in the spleen of chickens infected with indicated viruses at 84 dpi by qPCR. Data are shown in dots with medians, each dot corresponding to an individual bird. The HVT load was significantly reduced by 272-fold in the absence of the TMRs (****, p<0.0001, Mann-Whitney test, two-tailed). (B) 10^7^ splenocytes were co-cultivated with 3×10^5^ CESCs and the number of plaques counted after 4 days of infection. Shown are plaque numbers as dots with the medians shown as horizontal bars, each dot corresponding to an individual bird (WT, n = 9; vΔTMR, n = 10). The HVT reactivation was significantly reduced by 50.7-fold in the absence of the TMRs (****, p<0.0001, Mann-Whitney test, two-tailed).

To assess if virus latency is impaired in the absence of the TMRs, we quantified the viral loads in the spleen, the main site of HVT latency (Fig 4A) [16]. At the termination of the experiment (84 dpi), viral load was significantly reduced by 272-fold in the absence of the TMRs when compared to the WT. To determine if reactivation is also affected by the absence of the TMRs, we compared the reactivation of WT and vΔTMR mutant virus from splenocytes *ex vivo*. Splenocytes were purified at 84 dpi, co-cultivated with chicken embryonic skin cells (CESCs), carefully removed and plaques counted at 4 dpi. HVT reactivation was significantly impaired (50.7-fold) in the absence of the TMRs compared to the WT (Fig 4B). Zero to 4 plaques were observed per well in the case of vΔTMR. Taken together, our data revealed that latency in the spleen is severely impaired in the absence of the TMRs, which also impacts the ability of the virus to reactivate.

### The absence of the TMRs drastically impairs virus transport to the skin and almost abolishes feather follicle infection

**Fig 5:**
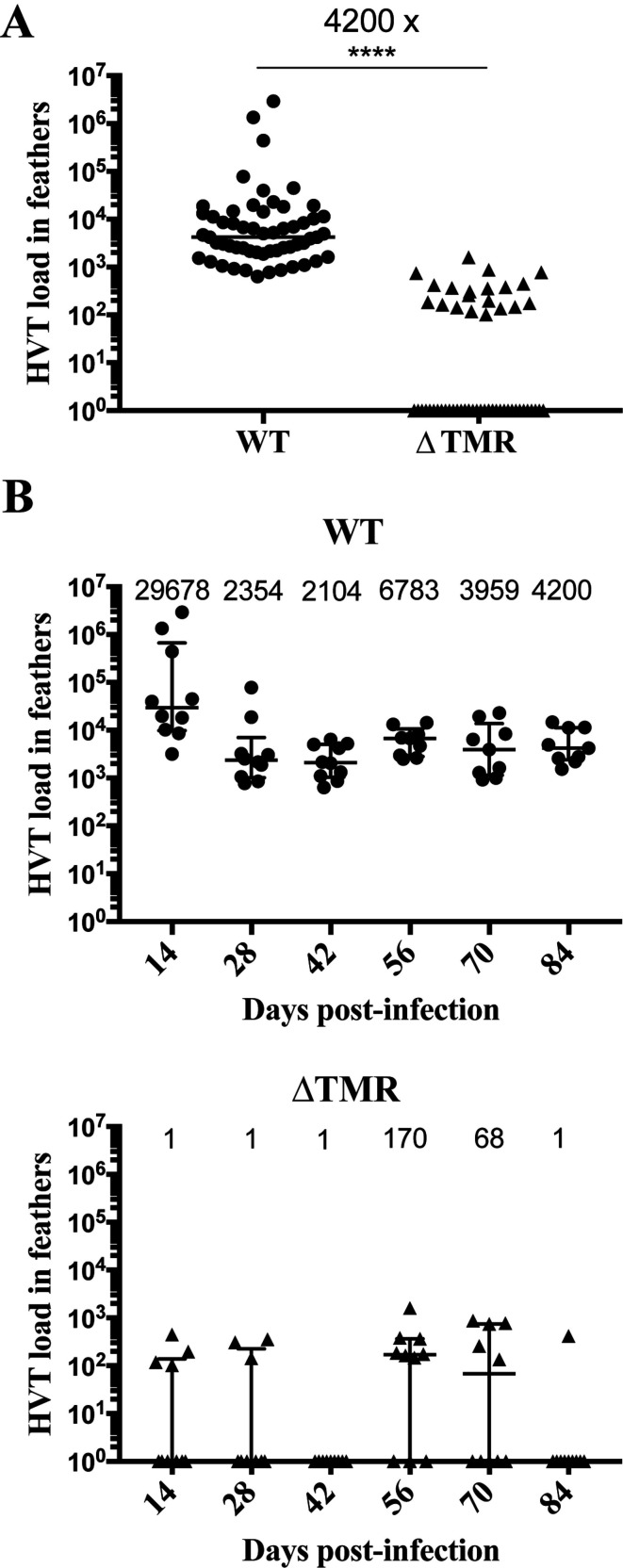
Viral load in the feathers is severely reduced in the absence of TMRs. (A) HVT genomes were quantified in feather tip material of chickens infected with indicated viruses and time points by qPCR. Data are shown in Tukey boxes with each sample as a dot. The HVT loads were significantly reduced by 4200-fold in the absence of the TMRs (Mann-Whitney test, p<0.0001). (B) Dynamic of HVT load over time. Data are shown in Tukey boxes as above with each HVT load visible as a dot and the median. The median loads were strongly reduced at all time points for vΔTMR (null at four time points) compared to WT. This difference between viruses was significant (p<0.0001, Wilcoxon test with a Holm correction for multiple comparisons).

During the course of infection, HVT is also transported to the FFE in the skin, from where it replicates and is shed into the environment [28,29]. We previously proposed that the persistence of the HVT vaccine in feathers may be due to the a frequent (re-)infection of FFE cells by latently infected cells, which mostly reside in the spleen [18]. To assess virus delivery to and replication in the skin in the context of reduced latency and reactivation, viral genome copies were measured by qPCR in feathers collected from birds at different time points during the experiment. Regardless of the time, viral loads of the vΔTMR virus in feathers were significantly reduced (4200-fold) compared to the WT (Fig 5A). 65.5% of the samples of the vΔTMR group had no detectable viral genome copies, while all of the WT samples were positive for the virus. Overall, this indicates that delivery of HVT to the skin is drastically impaired in the absence of the TMRs.

## Discussion

In this study, we set out to decipher the role of the TMRs in HVT integration, genome maintenance, and reactivation *in vitro* and *in vivo*. TMR sequences have been identified at the ends of 17 of the 83 full-length herpesvirus genomes [23], but their role remains elusive for almost all of them. Previous studies demonstrated that the TMRs in the genome of the highly oncogenic MDV facilitate its integration into host telomeres [21,26]. This integration is important for viral latency, reactivation and lymphoma development [21,30]. In case of HHV-6A, the TMRs play an important role in virus genome integration into host telomeres [24], which ensures maintenance of its genome in latently infected cells. Since HVT also possesses TMRs at both ends of the genome, we set out to assess their role in the life cycle of this important vaccine virus.

First, we generated an HVT platform virus that harbors a deletion of most of the internal repeat region, leaving only short terminal homologous sequences behind (ΔIR_LS-HR_) (Fig 1A). The remaining homologous sequences facilitated a rapid restoration of the deleted region (Fig 1C), which is consistent with data obtained for a similar deletion in MDV [27]. This ΔIR_LS-HR_ virus serves as an excellent platform for the rapid manipulation of the repeat regions in the HVT genome, as only a single locus has to be modified. It is crucial to emphasize that since all TMRs were removed from the vΔTMR virus genome, the virus cannot reacquire these sequences during the restoration of the deleted IR_LS-HR_ through recombination with the TR region. This platform virus will aid in the characterization of viral genes in the repeat regions and the generation of vector vaccines harboring foreign antigens in this region.

Based on this platform virus, we deleted the remaining TMR copy in the HVT genome (vΔTMR). Each a-like sequence in the HVT genome contains one TMR. In contrast, the oncogenic MDV contains two TMRs, the multiple telomeric repeats (mTMRs) and short telomeric repeats (sTMRs). Our data revealed that HVT efficiently replicates in the absence of its TMRs (Fig 1D and 1E). Replication was even increased compared to the WT, as observed to varying extents for HHV-6A and MDV (lacking the mTMRs) [21,24]. In case of MDV, the second TMR array (sTMRs) is required as a spacer between the cleavage and packaging signals [30]. MDV with mutated sTMRs replicated efficiently, while its deletion abrogated virus replication [30]. Overall, this highlights that the HVT TMRs resemble the function of the mTMRs in the MDV genome as both are dispensable for virus replication, but are crucial for integration.

Next, we established an *in vitro* integration assay using chicken T-cells, based on the recently published system for MDV [26]. Strikingly, HVT efficiently integrated into the ends of T-cell chromosomes (Fig 2A–2C), even at higher levels than previously observed for MDV [26]. Even though integration occurs efficiently, it remains challenging to assess this for latently infected cells *ex vivo* by FISH. This is due to the very low number of latently infected cells in the spleen and other lymphoid organs. A previous study attempted to detect these rare cells and could find a few harboring the integrated HVT genome [31].

Using this *in vitro* integration assay, we could investigate the role of the TMRs in the integration process.

In the absence of the TMRs, HVT integration frequency and maintenance of viral genomes were significantly impaired (Fig 2B and 2C). Interestingly, we observed that the vΔTMR genome was not present at the ends of the chromosomes containing the telomeres, but elsewhere in the host chromosomes. This is consistent with previous findings for MDV and HHV-6 lacking their TMRs) [21,24]. In the case of MDV, this random integration even occurred as a concatemer [21]. Considering i) the state of the cell cycle during integration, ii) the potential integration mechanisms and iii) how rapidly the virus integrates, it becomes clear that not every progeny cell in a population will harbor the virus genome. If integration occurs between S and M phase, then the virus can only integrate into one of the chromatids and therefore only 50% (or less) of the progeny cells will harbor the virus. If integration occurs single stranded (e.g. via strand invasion), then again only half of the progeny will harbor the integrated virus. In addition, if integration does not happen within the first cell cycle after infection, a portion of the resulting daughter cells will not harbor the virus. An integration frequency ranging from 20% to 50% would be considered highly effective. This is consistent with the integration frequency of 20% and 30% in progeny cells in vitro as observed for HHV-6 and MDV infection [24,26]. Taken together, the data revealed that HVT efficiently integrates into the ends of host chromosomes and that the process is dependent on its TMR sequences.

Next, we assessed the role of the TMRs (and, in turn, integration) *in vivo*. Our data revealed that the virus genome copies were significantly reduced in PBMCs in the absence of the TMRs (Fig 3A and 3B). Given the overall very low levels of virus genome copies in the vΔTMR group, certain time points were below the detection limit (13.2%). This does not mean that these birds were virus negative, but rather that the viral loads in the blood were below the detection limit. A reduction in the viral load in the blood was also previously observed for MDV lacking its TMRs [21], but to a lesser extent. The observed differences could be attributed to the different measurement methods, as virus loads for MDV were assessed in total blood, whereas those for HVT were determined in PBMCs. Interestingly, the virus loads in PBMCs were most drastically reduced at later time points (from 42 dpi) with some animals having no detectable virus genomes (Fig 3B). At late time points (after 14 dpi), HVT already established latency and the reduced viral loads likely reflect a reduction in latently infected cells that could also transport the virus to the skin.

To further assess these aspects, we investigated the viral levels in the spleen of HVT-vaccinated animals, the main site of latency. The viral loads detected in the spleens of vΔTMR-infected animals were significantly reduced (272-fold reduction), indicating that efficient integration of the viral genome into the host chromosomes is required for efficient genome maintenance during latency, as observed in T-cells *in vitro*. As the MDV TMR mutant viruses were severely impaired in their ability to reactivate [21], we assessed the reactivation of the virus in splenocytes. Consistent with the diminished levels in the spleen, reactivation was significantly reduced (Fig 4A). These lower reactivation levels could also be due to the reduced viral genome maintenance in splenocytes. Interestingly, recombinant HVT vaccines (such as HVT-ND) are capable of providing long-term protection with a single vaccine dose [13], which could be due to frequent reactivation and re-exposure to the immune system. The HVT vΔTMR mutant might help to address this aspect in future studies.

Finally, we assessed if the transport to the FFE is affected in the absence of the TMRs. Viral loads were drastically reduced in feathers of animals infected with vΔTMR (Fig 5A). Interestingly, the virus was not detected continuously in the feathers in the absence of the TMRs. The disappearance (and reappearance) of the virus in the feathers suggests that the feathers are regularly reinfected from a reservoir e.g. the spleen as previously hypothesized [18]. This could explain why HVT is detected in the feathers for a very long time post-vaccination, rather than persisting in each feather follicle for a life time. We propose two hypotheses to explain the occurrence of reinfection. The first hypothesis suggests that HVT may reactivate permanently in the spleen, and the reactivated lymphocytes may subsequently enter the feather follicles. The second hypothesis proposes that latent virus-infected lymphocytes may circulate in the body, and the virus may reactivate upon reaching the feather follicles. Based on current knowledge, the second hypothesis appears to be the most plausible as there is no known instance of a herpesvirus permanently reactivating at its sites of latency. Future studies should address these aspects to understand the contribution of reactivation to vaccine protection and shedding and to improve the control of virus shedding from the FFE into the environment.

**Fig 6:**
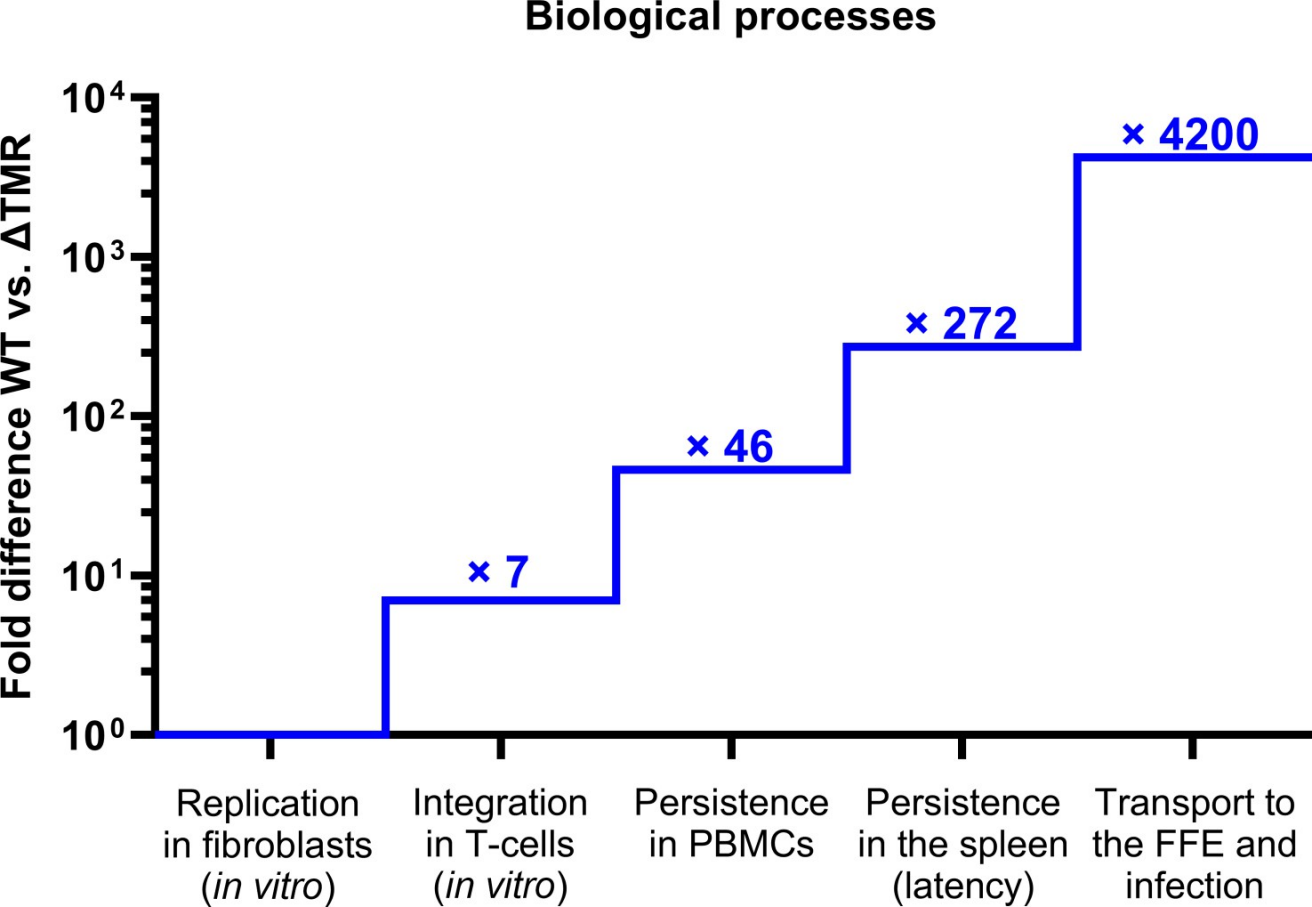
Schematic representation of the successive steps followed by HVT vaccine integration into the host. The numbers indicate the reduction measured in our study for the vΔTMR mutant vs WT at each consecutive step of the HVT replication cycle. Note the escalating impairment from one step to the next.

Our study provides evidence that the TMRs play a role in different stages of the HVT replication cycle. Importantly, the effect of the TMR deletion is more pronounced *in vivo* than *in vitro*. *In vivo*, the deletion progressively hinders biological processes at each step–ultimately almost abolishing FFE infection (Fig 6). In conclusion, our study provides important evidence that the HVT vaccine efficiently integrates into the chromosomes of T-cells and that the TMRs play an important role in this process. Integration is also crucial for the efficient persistence of the virus in the host and its delivery to the skin, where it is shed into the environment. Along with the diminished virus latency in the spleen virus, reactivation was significantly reduced both *in vitro* and *in vivo*. Overall, our findings highlight that HVT efficiently integrates and that the TMRs play a crucial role in the integration of this important vaccine virus.

## Materials and Methods

### Ethics statement

The *in vivo* experiment was carried out according to the guidance and regulation of the French Ministry of Higher Education, Research and Innovation (MESRI) with appropriate staff, good animal practices, and project authorizations (protocol number APAFIS #19096). As part of this process, the experimental protocol was thoroughly examined and approved by the appropriate local ethics committee, CREEA VdL (“Comité d’Ethique pour l’Expérimentation Animale Val de Loire”).

### Cells

Chicken embryonic cells (CECs) were prepared from VALO SPF 11-day-old embryonated chicken eggs (Valo BioMedia; Osterholz-Scharmbeck, Germany) as described previously [32]. CECs were cultured in Eagle’s minimal essential medium (MEM; PAN Biotech, Germany) supplemented with 10% fetal bovine serum and antibiotics (100 U/mL penicillin and 100 μg/mL streptomycin). CESCs were prepared from WL B19 SPF 12-day-old embryonated chicken eggs (INRAE) as described previously [33] and cultivated in William’s modified E medium with 2% chicken serum, 3% fetal calf serum (FCS). The chicken T-cell line 855–19 was propagated in RPMI 1640 media (PAN Biotech, Germany) supplemented with 1.5% sodium pyruvate, 1.5% nonessential amino acids, 10% FBS, and 1% penicillin/streptomycin, and maintained at 41°C in a 5% CO_2_ atmosphere.

### Generation of recombinant viruses

HVT lacking the TMRs (vΔTMR) was generated using the HVT bacterial artificial chromosome (BAC) encoding an enhanced green fluorescent protein (eGFP) in the mini-F cassette (a kind gift from V. Nair, The Pirbright Institute, UK) [34]. First, we deleted most of the internal repeat regions (HVT ΔIR_LS-HR_), retaining only the ends of the IR_L_ and IR_S_ regions (0.9 and 1.2 kbp, respectively), as previously described for MDV [27] (Fig 1) using two-step Red-mediated mutagenesis [35]. This deletion is rapidly restored upon reconstitution and facilitates a rapid manipulation of the repeat regions using mutagenesis [27,36]. Next, the TMRs were deleted in HVT ΔIR_LS-HR_, resulting in an HVT mutant lacking all TMRs (vΔTMR). All recombinant mutants were confirmed by RFLP, Southern blotting, PCR, Sanger-, and Illumina MiSeq NGS sequencing (Table 1).

**Table 1:** Primers and probes.

Construct name		Sequence (5’ → 3’)
EP PCR ΔIR_LS_	for	GCCTTTTATCGCATTTCATTCGAGAGCGATGACATGCGGG GGTGCCACCGCCCGCATAGGTAGGGATAACAGGGTAATCGATTT
	rev	GATATGGTGTGATATGAATGCCTATGCGGGCGGTGGCACC CCCGCATGTCATCGCTCTCGGCCAGTGTTACAACCAATTAACC
EP PCR ΔTMR	for	GGGTACCTGTTAACCCCGGGGGTATAAATTGAGGGGGGGG GTTAGTTTTTTTTTCTGCAATAGGGATAACAGGGTAATCGATTT
	rev	ACGACTCTCCCGGCCGCGCATTGCAGAAAAAAAAACTAAC CCCCCCCCTCAATTTATACCGCCAGTGTTACAACCAATTAACC
Sequencing of ΔIR_LS_	for	GGACTGTAAAAACTGACAAATGCG
	rev	CGTCAACAACGACTAACCACGC
Sequencing of ΔTMR	for	GCCGAGGGAAAACAGGTC
	rev	CCTCCAGAGCACACTCCTT
SORF1 (qPCR)	for	GGCAGACACCGCGTTGTAT
	rev	TGTCCACGCTCGAGACTATCC
	probe	FAM-AACCCGGGCTTGTGGACGTCTTC-TAMRA
Detection of ΔIR_LS_ (qPCR)	for	CCGGCGATACAATTTGCAC
	rev	TTTCATTCGAGAGCGATGAC
	probe	FAM-AGCTGCGCGAACCATCAATGGGC-TAMRA
ICP4 (qPCR)	for	TCTTGCACCGAGATGATCGAT
	rev	AAAATACCATAGATTCGAGAGGTTCAG
	probe	FAM-AAATCCACCCGTCGAGTCGCCC-TAMRA
iNOS (qPCR)	for	GAGTGGTTTAAGGAGTTGGATCTGA
	rev	TTCCAGACCTCCCACCTCAA
	probe	FAM-CTCTGCCTGCTGTTGCCAACATGC-TAMRA
TMR DIG-labeled probe		DIG-TTAGGGTTAGGGTTAGGGTTAGGGTTAGGGTTAGGGTTAGGG
FISH PCR probe 1	for	GTTCGGACGTTTCGGTTTTC
	rev	GTAAACCAGCGAGACGCTAA
PCR-based probe 2	for	AAAGATACGCATGGGCTGAG
	rev	AATTCGTCAAATCGGGCGTA
PCR-based probe 3	for	GTTGCATATGCGTAAGTCGC
	rev	CCCCAATCCCATGGTCAAAA
PCR-based probe 4	for	GTTAGCAACACAGGTCCCAA
	rev	ATGTCATCAACCCTACCCCA
PCR-based probe 5	for	GGCGTGATCCTCTAGCAAAA
	rev	GCTACATCACGCAAGACTGA
PCR-based probe 6	for	GGCGTCTTTCTCGAAGATGT
	rev	GACAGGCGCTATATTCCTCG

EP, *en-passant* mutagenesis; for, forward primer; rev, reverse primer; sequences used for the first homologous recombination are underlined

### Virus reconstitution and propagation

All recombinant viruses were reconstituted by calcium transfection of CECs with purified BAC DNA as described previously [37]. eGFP-expressing viruses were used for *in vitro* characterizations. For the animal experiment, the mini-F cassette was removed by co-transfection of the BAC DNA and the pCAGGS-NLS/Cre plasmid [36]. The BAC removal was verified by the loss of GFP. Viruses were propagated on CECs for 3 to 5 passages, virus stocks were frozen, titrated, and stored in liquid nitrogen.

### Plaque size assays

The replication properties of the recombinant viruses were assessed using plaque size assays as described before [38]. Briefly, CECs were infected with 100 pfu of the different viruses. At 6 dpi, cells were fixed with 4% paraformaldehyde for 20 min and washed with PBS. The area of at least 50 randomly imaged plaques was determined using the Image J software (NIH) in a blinded manner.

### Multi-step growth kinetics

Replication properties of recombinant viruses were determined by multi-step growth kinetic as previously described [38]. Briefly, CECs were infected with 100 pfu of indicated viruses. Viral genome copies were measured over six days by quantitative PCR (qPCR) using primers and probes specific for the HVT-infected cell protein 4 (ICP4) and the chicken inducible nitric oxide synthase (iNOS) genes (Table 1). ICP4 copies were normalized against the cellular iNOS copies.

### Restoration assays

To detect the restoration of the deleted repeat sequences, restoration efficiency was assessed as described previously [27]. Briefly, upon transfection, the viruses were amplified for 15 passages. During each passage, infected cells were trypsinized, split at a 1:100 ratio, and subsequently mixed/incubated with uninfected CECs. Additionally, at passages 1, 5, 10, and 15, samples of infected cells were harvested for DNA extraction using the Quick-DNA Viral Kit (ZYMO Research, CA, USA). To ascertain the restoration of deleted repeat regions, we performed qPCR using specific primers and probes to quantify the number of genomes that still retained the deletion site. These copy numbers were then normalized against DNA copies of the viral SORF-1 gene (Table 1).

### Southern blotting

To confirm the TMR deletions, BAC DNA was digested with BamHI and separated on an agarose gel. Southern blotting was performed after the transfer of digested BAC DNA onto a positively charged nylon membrane (Immobilon-NY+, Merck Millipore, Darmstadt, Germany). Fragments containing the TMR arrays were detected with a TMR-specific DIG-labeled probe (Table 1), an anti-DIG alkaline phosphatase-labeled antibody (Roche GmbH, Mannheim, Germany), and the CDP-*Star* ready-to-use detection system (Roche GmbH, Mannheim, Germany).

### *In vitro* HVT integration assays

To determine the integration efficiency of HVT, we established a cell-based integration assay based on the recently published system for MDV [26]. Briefly, the chicken T-cell line 855–19 was infected by co-cultivation with a highly infected CEC monolayer [39]. After 16 h, T-cells were carefully removed and seeded into a new cell culture dish. The percentage of infected T-cells (GFP-reporter) was measured by FACS using the CytoFlex S flow cytometer (Beckman Coulter, Brea, CA, USA). Infected T-cells were cultured for up to 14 days. FACS analysis was also used to confirm latent infection of cells analyzed at 14 dpi, where all cells were GFP-negative. Viral genome copies were analyzed by qPCR relative to cellular genome copies using specific primers and a probe for HVT ICP4 and the cellular iNOS gene (Table 1). Integration of HVT was visualized in metaphase chromosomes at 14 dpi with a set of PCR-based HVT-specific probes by FISH as described previously (Table 1) [26,40]. Reactivation of the integrated viruses in latently infected T-cells was assessed as described previously [21,26]. Briefly, the GFP-negative T-cells harboring the latent virus genome were serum starved, seeded on CEC monolayers and incubated at room temperature for 1h. T cells were then co-cultured with CECs overnight, carefully removed and the number of plaques in the CEC monolayers quantified after 4 dpi.

### Animal experiment

Twenty specific pathogen-free (SPF) White Leghorn chickens (B13/B13 haplotype) were obtained from the INRAE animal facility. One-day-old chicks were inoculated subcutaneously with 5000 pfu of either HVT WT (*n* = 10) or vΔTMR (*n* = 10) and the virus inoculums were back-titrated. The two groups were kept in two independent isolation units for twelve weeks. Whole blood and 2–3 growing feathers were collected from all chickens at 14, 28, 42, 56, 70, and 84 dpi. At the end of the experiment, all chicks were humanely euthanized, necropsied, and the spleens harvested in 10 ml of Iscove medium at 4°C to quantify the virus load and reactivation (see below). One animal from the WT group died after blood sampling at week 6 and did not contribute data thereafter.

### Quantification of HVT genomes in the chicken PBMCs, feathers, and splenocytes

DNA was extracted from PBMCs, growing feather tips (proximal ends containing feather pulp and epithelium) and splenocytes using the QIAamp DNA mini kit (Qiagen, Germantown, MD, USA) as described previously [18,41]. PBMCs and splenocytes were purified on a Ficoll-based lymphocytes separation medium (CMSMSL01-01, Eurobio Scientific, Les Ulis, France) as previously reported [41]. HVT genome copies were quantified by qPCR as reported previously [18] and reported relative to one million cells. Data obtained from triplicate measurements were designated as non-interpretable (Ni) if there were variations greater than 0.5 in at least two replicates or if only one of the replicates had a quantification cycle (Cq) value obtained. This was confirmed in two independent qPCR runs using the same DNA sample. It is worth noting that all the results obtained from the iNOS qPCR were interpretable, indicating good-quality DNA. The Ni data were exclusively observed in the qPCR targeting HVT. All individual viral loads are available as supplemental information (S1 Table).

### HVT reactivation from splenocytes

Half of each spleen harvested at final necropsy (84 dpi) was dissociated and cells sedimented for 10–20 min. Cells in the supernatant were harvested by centrifugation. The splenocytes were purified via gradient centrifugation on lymphocyte separation medium (CMSMSL01-01, Eurobio Scientific), washed, and counted. To ensure optimal plaque countability, five-fold serial dilutions were performed in Iscove medium with 2% chicken serum and 3% FCS starting from 10^7^ splenocytes per well. The splenocytes were added to CESC monolayers (~300,000 cells) in 12-well-plates. After 18 h, the splenocytes were removed, the CESC monolayers were extensively washed and cultivated in William’s modified E medium supplemented with 1% chicken serum, 1.5% FCS, penicillin, streptomycin and hexamethylene bisacetamide (1 mg/mL). After 4 dpi, the cells were fixed with 4% paraformaldehyde. Plaques were detected using an HVT-specific antiserum and a goat anti-chicken Alexa Fluor 488 secondary antibody (Molecular Probes). The number of plaques was counted using an Axiovert 200M inverted epifluorescence microscope (Zeiss) with a 5x Fluar long-distance objective.

### Statistical analyses

Statistical analyses were performed using GraphPad Prism (v. 7; GraphPad Software, Inc., USA) and the R software (v.3.4.3.). Non-parametric tests were used for the data analysis of the *in vivo* experiment, because of small sample sizes (9–10 independent subjects per virus). All common applied statistical tests are indicated in the respective figure legend. For the analysis of variance in case of mixed models (e.g. several viruses at different dates), a non-parametric ANOVA-like test for non-parametric analysis of longitudinal data in factorial experiments using ranks and adjusted p-values for pairwise comparisons was used [42,43]. Data were considered significantly different if p≤ 0.05. The number of repeats is indicated in respective figure legends.

## Supporting information

S1 Table HVT load in PBMCs, feathers and spleen. (PDF)
