# Supplementary material for: Impact of viral telomeric repeat sequences on herpesvirus vector vaccine integration and persistence
Source: PLoS Pathog. 2024 May 28;20(5):e1012261. doi: 10.1371/journal.ppat.1012261 (PMC11161090; doi:10.1371/journal.ppat.1012261)
Supplement: S1 Table — (PDF) [file ppat.1012261.s001.pdf]

Table S1. HVT load in PBMCs, feathers and spleen.

			HVT load		
**chickID**	**HVT**	**dpi**	**PBMCs**	**feathers**	**spleen**
77	WT	14	487	439422	
78	WT	14	954	10276	
79	WT	14	*NA	39623	
80	WT	14	740	18174	
81	WT	14	1595	44902	
82	WT	14	387	19732	
83	WT	14	651	3185	
84	WT	14	688	2931140	
85	WT	14	722	8642	
86	WT	14	NA	1339749	
91	ΔTMR	14	72	449	
92	ΔTMR	14	65	1	
93	ΔTMR	14	NA	1	
94	ΔTMR	14	NA	102	
95	ΔTMR	14	31	1	
96	ΔTMR	14	46	1	
97	ΔTMR	14	19	197	
98	ΔTMR	14	23	119	
99	ΔTMR	14	36	1	
100	ΔTMR	14	55	1	
77	WT	28	659	78227	
78	WT	28	1848	1065	
79	WT	28	986	3062	
80	WT	28	1707	1875	
81	WT	28	2400	18656	
82	WT	28	2662	2537	
83	WT	28	2033	849	
84	WT	28	1680	3220	
85	WT	28	1094	776	
86	WT	28	1389	2171	
91	ΔTMR	28	122	1	
92	ΔTMR	28	96	**Ni	
93	ΔTMR	28	Ni	1	
94	ΔTMR	28	35	1	
95	ΔTMR	28	34	1	
96	ΔTMR	28	NA	143	
97	ΔTMR	28	68	1	
98	ΔTMR	28	Ni	358	
99	ΔTMR	28	27	1	
100	ΔTMR	28	91	306	
77	WT	42	1630	639	
78	WT	42	6368	5217	
79	WT	42	3078	1109	

80	WT	42	4336	863
81	WT	42	4702	4202
82	WT	42	5906	5047
83	WT	42	4970	1322
84	WT	42	5194	6372
85	WT	42	3100	2048
86	WT	42	5548	2159
91	ΔTMR	42	279	1
92	ΔTMR	42	104	1
93	ΔTMR	42	73	1
94	ΔTMR	42	1	1
95	ΔTMR	42	1	1
96	ΔTMR	42	94	1
97	ΔTMR	42	192	1
98	ΔTMR	42	1	1
99	ΔTMR	42	39	1
100	ΔTMR	42	58	Ni
77	WT	56	1838	2993
78	WT	56	6483	14329
79	WT	56	2847	2496
80	WT	56	4988	6783
81	WT	56	4505	2641
82	WT	56	9237	6930
83	WT	56	3705	13245
84	WT	56	NA	NA
85	WT	56	3647	4674
86	WT	56	9032	8116
91	ΔTMR	56	182	1
92	ΔTMR	56	216	364
93	ΔTMR	56	1	184
94	ΔTMR	56	92	1
95	ΔTMR	56	170	380
96	ΔTMR	56	Ni	1615
97	ΔTMR	56	228	164
98	ΔTMR	56	1	175
99	ΔTMR	56	144	147
100	ΔTMR	56	93	1
77	WT	70	1933	921
78	WT	70	4897	6433
79	WT	70	2200	8412
80	WT	70	6276	19185
81	WT	70	5349	1000
82	WT	70	14145	3959
83	WT	70	5899	22991
84	WT	70	NA	NA
85	WT	70	4628	1300

86	WT	70	5038	1636	
91	ΔTMR	70	268	1	
92	ΔTMR	70	141	**135**	
93	ΔTMR	70	62	1	
94	ΔTMR	70	66	776	
95	ΔTMR	70	103	**260**	
96	ΔTMR	70	254	742	
97	ΔTMR	70	42	1	
98	ΔTMR	70	Ni	1	
99	ΔTMR	70	41	1	
100	ΔTMR	70	69	875	
77	WT	84	678	11400	6816
78	WT	84	3050	4940	28802
79	WT	84	1100	2560	7484
80	WT	84	3840	2780	40114
81	WT	84	2240	4200	17692
82	WT	84	1580	2230	21556
83	WT	84	3420	14800	26725
84	WT	84	NA	NA	NA
85	WT	84	2000	11200	17588
86	WT	84	2260	1550	14783
91	ΔTMR	84	10	1	88
92	ΔTMR	84	58	417	233
93	ΔTMR	84	22	1	481
94	ΔTMR	84	36	1	62
95	ΔTMR	84	1	1	**1**
96	ΔTMR	84	26	1	26
97	ΔTMR	84	55	1	67
98	ΔTMR	84	78	1	22
99	ΔTMR	84	1	1	**1**
100	ΔTMR	84	18	1	134

*NA: no DNA sample **Ni: not interpretable
